# DenseNet_ HybWWoA: A DenseNet-Based Brain Metastasis Classification with a Hybrid Metaheuristic Feature Selection Strategy

**DOI:** 10.3390/biomedicines11051354

**Published:** 2023-05-04

**Authors:** Abdulaziz Alshammari

**Affiliations:** Information Systems Department, College of Computer and Information Sciences, Imam Mohammad Ibn Saud Islamic University (IMSIU), Riyadh 11432, Saudi Arabia; aashammari@imamu.edu.sa

**Keywords:** brain metastases, classification, neural network, MRI, optimization, feature selection

## Abstract

Brain metastases (BM) are the most severe consequence of malignancy in the brain, resulting in substantial illness and death. The most common primary tumors that progress to BM are lung, breast, and melanoma. Historically, BM patients had poor clinical outcomes, with limited treatment options including surgery, stereotactic radiation therapy (SRS), whole brain radiation therapy (WBRT), systemic therapy, and symptom control alone. Magnetic Resonance Imaging (MRI) is a valuable tool for detecting cerebral tumors, though it is not infallible, as cerebral matter is interchangeable. This study offers a novel method for categorizing differing brain tumors in this context. This research additionally presents a combination of optimization algorithms called the Hybrid Whale and Water Waves Optimization Algorithm (HybWWoA), which is used to identify features by reducing the size of recovered features. This algorithm combines whale optimization and water waves optimization. The categorization procedure is consequently carried out using a DenseNet algorithm. The suggested cancer categorization method is evaluated on a number of factors, including precision, specificity, and sensitivity. The final assessment findings showed that the suggested approach exceeded the authors’ expectations, with an F1-score of 97% and accuracy, precision, memory, and recollection of 92.1%, 98.5%, and 92.1%, respectively.

## 1. Introduction

The most prevalent cerebral tumors are brain metastases (BMs), which frequently develop from aggressive melanoma, breast cancer, and lung cancer [1]. Due to more comprehensive therapeutic options and lung cancer monitoring initiatives in many nations, cancer patients are living longer, which has increased the incidence of this disease. The diagnosis of BMs relies heavily on the use of contrast-enhanced T1-weighted imaging (CET1WI) magnetic resonance (MR) scans, which are also employed for long-term monitoring to gauge therapy effectiveness [2]. The majority of patients have three or fewer brain tumors when they first appear, although 40% of patients have more than three. Despite the fact that identifying BMs is a laborious and time-consuming physical process for radiologists, they are very important in the preliminary diagnosis of tumors, definition of the initial tumor volume, and monitoring of volume fluctuations as a result of therapy [3]. The early and precise identification of BMs is essential for proper therapy planning, as the existence of BMs can shift overall oncologic management. To aid physicians, deep learning-based techniques have recently been proposed for autonomous spotting or segmenting of BMs in MRI images [4]. The fact that BM and other structures, such as cerebral arteries, share comparable physical characteristics, and that BMs vary greatly in size and spread, renders this a difficult task [5]. Recently, many researchers have introduced multi-sequence MRI automated recognition and segmentation methods circumvent the constraints of using only MRI sequences. According to this perspective, precise BM identification and differentiation from various suspect areas (BM imitators) are crucial for effective evaluation and treatment [6]. Early and precise detection of the cause(s) of BM prior to surgery could alter individual treatment strategies, which is of great therapeutic significance. The epidermal growth factor receptor (EGFR) [7] gene mutant state is critical for treatment plans, such as EGFR-tyrosine kinase inhibitor (EGFR-TKIs) administration, for NSCLC patients with BMs. The HER2 condition is essential to deciding on treatment approaches for BC patients with BMs. This is due to the fact that patients who are HER2-positive frequently receive tailored antibody treatments and typically have poor prognoses [8]. Since it may not always be practically feasible to obtain tissue from the main tumor, the spread may serve as a valuable backup source of information regarding the features and gene status of the primary tumor. However, due to the lack of particular indicators, radiologists are hardly ever able to determine the spread sources or examine MRI images visually to determine the gene state of the primary tumor. Artificial intelligence (AI) and computer-aided diagnostic (CAD) developments are becoming ever more significant in the area of medical imaging [9]. The term ‘radiomics’ refers to the methodical calculation and study of a significant number of numeric characteristics used in medical imaging. However, few investigations have used CAD to identify the spreading cause of BM. In earlier studies, it was assumed that the total brain spread of tumor volume was uniform. However, recent studies have shown that solid tumors can be diverse, with several tumor areas being more physiologically invasive and possibly reflecting various biological processes [10]. Intra-tumor heterogeneity, or ITH, has been recognized for having important ramifications that represent unique tumor development. To segment the entire tumor area into intra-tumor sub-regions and enable the collection of useful information from the sub-regions, sub-region-based radiomics methods have been proposed. According to statistics, sub-region radiomics studies have been carried out in cases of esophagus carcinoma, lung cancer, and breast cancer, and have demonstrated the potential to greatly enhance the diagnostic performance of radiomics techniques [11]. To the authors’ understanding, brain cancer has not been studied using sub-region radiomics. Therefore, the following are the accomplishments of this work:

This simplified form of the U-Net can be readily implemented on devices with limited (processing and memory) resources. This research is novel in several ways, as it employs the shallowest version of U-Net on a large dataset to derive characteristics that improve the BP forecast process.

The method known as HybWWoA, which combines the water waves optimization and whale optimization techniques, is used to choose the features by reducing the size of the recovered feature.

DenseNet was developed to use dense connections from its backbone design to identify BM MRI images with high precision and accelerated learning.

The remainder of this article is divided into the following sections: Section 2 discusses a few previous research studies, Section 3 illustrates the suggested methodology and approach, Section 4 presents the results of the experiment and discussion, and Section 5 includes a conclusion and suggestions for future research.

## 2. Related Works

Traditional neural networks (NN) have been expanded by the DL structure, which extends the input and output levels’ hidden layers to show more complicated and irregular connections. Due to its astounding efficacy, this concept has attracted academic interest in recent years as an optimal answer to numerous issues in medical image analysis applications.

According to the author of [12], existing systems have been effective in pinpointing the precise tumor area and concealed border features with the least amount of computational complexity. In addressing these issues, the Harris Hawks optimized convolution network (HHOCNN) has been employed in this study. To reduce the rate of erroneous tumor identification, noise pixels were removed from the brain magnetic resonance imaging (MR) images. The tumor area was consequently determined using the potential region method. By reducing the size of the recovered features, the Improved Chimpanzee Optimization Algorithm (EChOA) was used in [13] to select the features. The feature categorization procedure was consequently carried out using the softmax algorithm and ResNet-152. According to [14], preparation and data enrichment techniques such as skewed data concepts have been developed to improve categorization rates. Additionally, AlexNet and VGG19 were used to carry out particular tasks. In order to classify brain tumors, all characteristics were finally combined into a unique feature vector. Several of the selected characteristics were discovered to be irrelevant for accurate categorization. In order to identify the most discriminative features and achieve the highest tumor categorization rate, this study used an effective feature selection method called “slap swarm” [15]. The suggested paradigm streamlines the naturally complicated technique for detecting brain tumors and improves performance. The HBTC framework received the raw brain MRI dataset after preprocessing and segmentation to identify the tumor region. In [16], an innovative AI optimization method, deep neuro-evolution (DNE) performs well with limited training data, making it ideal for use by radiologists. This study utilized a DNE parameter search in order to improve a convolutional neural network (CNN) model that forecasts the progression or regression of an intrusive brain illness. SCENIC (Separable Convolution Enabled Non-Invasive Classification) is a hardware–software co-design technique developed in [17] for the identification and classification of glioma brain tumors. Convolutional neural networks are used in [18] to create a multi-classification system for brain lesions, achieving early detection. Doctors and therapists can verify their original screening for the purpose of categorizing various types of brain lesions using the recommended CNN models. In [19], OTSU and adaptive particle swarm optimization both helped to determine the ideal cutoff value. Regarding brain MRI, anisotropic diffusion filtration is used to reduce noise and enhance image clarity. In [20], deep neural networks were used to classify MRS data into two groups: tumors and healthy tissue with artificial lesions. To properly classify MRS data, it is recommended to use a multi-layer model consisting of Long Short-Term Memory (LSTM) and Bidirectional Long Short-Term Memory (Bi-LSTM) deep neural networks. The deep learning VGG16_CNN model is proposed to diagnose brain tumors in MR images [21]. An expert radiologist can use this method to classify brain MRIs into meningiomas, gliomas, and pituitary tumors by clinically differentiating BMs.

The primary issue with using selected neural networks to classify and split MRI images is the large volume of images within the collection. Furthermore, if all available aircraft were used, the collection could increase, as MRI images are acquired in a variety of aircraft. It is important to carry out image pre-processing prior to feeding the images into the neural network because overfitting frequently has an effect on the categorization results. This collection is a significant fraction of the size of the databases that are routinely employed in the field of artificial intelligence, despite still being considered substantial compared to other MRI image datasets that are currently available. This study aimed to demonstrate that the minimized design could perform effectively in comparison to more sophisticated ones. Using a simple network requires less time and money for installation and training, which is of critical concern because there are resources available for clinical diagnosis smartphone devices. The technology must be broadly adaptable in order to be used in routine clinical diagnostics. In order to demonstrate how the subject-wise cross-validation method yields more accurate findings for future applications, this study also aimed to investigate the network’s ability to generalize for clinical research.

## 3. System Model

**Figure 1:**
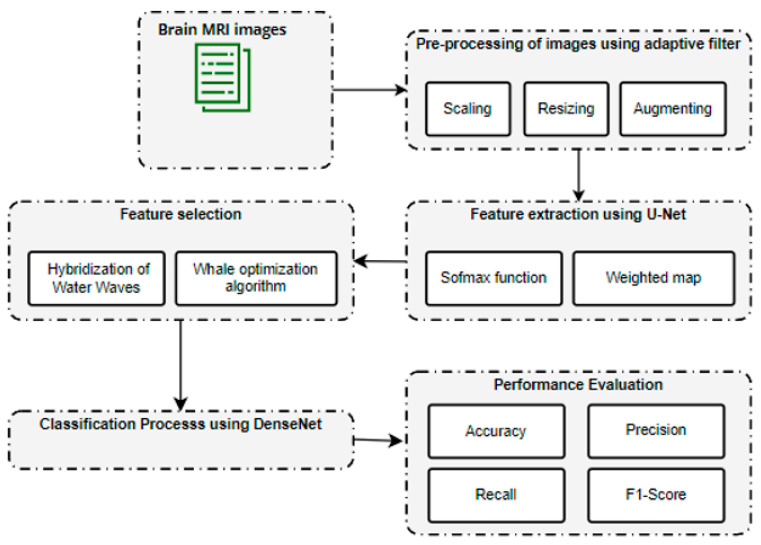
Overall architecture for brain metastases classification.

The overall block diagram for the proposed brain metastases classification using the brain tumor dataset is shown in Figure 1.

The initial sample consisted of 3064 T1-weighted contrast-enhanced images of brain tumors. These data were pre-processed using an adaptive filter. Consequently, feature extraction was carried out using U-Net. After feature extraction, a hybridization of Water Waves and the Whale Optimization Algorithm was utilized to select a limited set of features. Finally, the tumor region was classified using DenseNet.

## 4. Dataset Description

**Figure 2:**
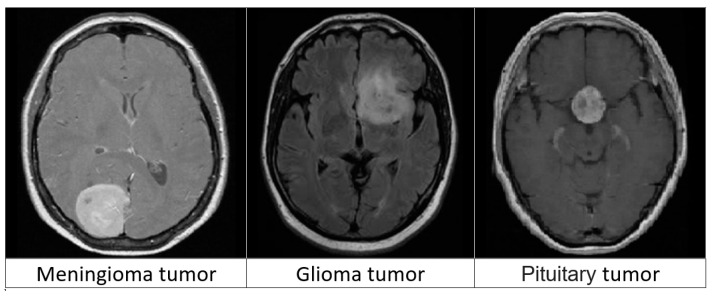
Three types of brain tumors from MRIs.

In this work, we used a dataset from publicly accessible online data provided by Cheng et al. [22]. The authors provided a public data set aimed at synthesizing images for deep learning frameworks and analyzing their efficiency in identifying brain tumors on MRIs. The dataset consists of 3064 T1-weighted contrast-enhanced MRI images from the image database from two medical facilities in China: the Nanfang Hospital and the General Hospital at the Tianjin Medical University. The images were obtained between 2005 and 2010, but were first published online in 2015. The images were obtained from 233 patients and cover three types of tumors, including pituitary tumors (930 images), meningioma (708 images), and glioma (1426 images). Each of these tumors from all three planes is portrayed in Figure 2. Furthermore, the images feature different planes: the axial plane (994 images), sagittal plane (1025 images), and coronal plane (1045 images).

## 5. Pre-Processing of Images

This study used several image pre-processing techniques before using the images for training or assessment. This study first supplemented images using zero-values because the source images had varying grid values. If required, the images were consequently scaled to fit into a 900 × 900 grid. The U-Net model required that the input images had a zero mean; therefore, this study subsequently conducted a per-image mean reduction to render all images zero-centered. The amount of our training data was consequently increased by using a left–right shift procedure as the final preparation phase. This study suggests a geographically adjustable soft truncation method with cutoff value determination based on local data and a hierarchy correlation map. The suggested method utilizes the wavelet coefficients’ intra-scale dependence by determining the signal’s local spread and the inter-scale dependence by determining the outlines based on the hierarchical correlation map. The algorithm’s results demonstrate that noise in images can be reduced while maintaining or even improving the clarity of outlines. To precisely pinpoint the positions of outlines or other significant features in the image, this study describes the direct spatial correlation of the uncertain wavelet transformation on a number of neighboring scales as shown in Equation (1):

(1) $${Corr}_{l}(m,n)=\prod_{i=0}^{l-1}w\left(m+1,n\right),n=1, 2,\ldots \ldots N$$

where w(j,n) are the wavelet coefficients of the deteriorated noise signal corresponding to scale j, l is the number of scales taken into account in straight multiplication, M is the overall number of scales, and N is the number of samples in the signal.

If this study considers an uncertain wavelet decomposition on three layers (giving three scales), then the direct spatial association will be as follows in Equation (2):

(2) $${corr}_{2}\left(1,n\right)=w\left(1,n\right)w\left(2,n\right)$$

As a function of $\left\{w\left(1,n\right)\right\}$, data representing ${\{corr}_{2}\left(1,n\right)\}$ are rescaled. By contrasting the exact values of ${corr}_{2}\left(1,n\right)$, the most significant outlines are found to be $w\left(1,n\right)$. In Equation (3), the location n is considered to be part of a curve if:

(3) $$\left|{corr}_{2}\left(1,n\right)\right|>\left|w\left(1,n\right)\right|$$

The value of $w\left(1,n\right)$ in the original matrix is deleted, and the value of the $w\left(1,n\right)$ coefficient corresponding to location n, marked as pertaining to the contour, is saved in a new matrix $w_{new}(1,n)$ along with the contour position. As a final step, all designated contours are pulled from ${corr}_{2}\left(1,n\right)$ and $w\left(1,n\right)$ by canceling their values in the places that were found to be contour locations. Consequently, this study obtains two new datasets, ${corr}_{2}\left(1,n\right)$ and $w^{\prime }\left(1,n\right)$, which serve as the foundation for the subsequent contour determination.

## 6. Feature Extraction Using U-Net

The unprocessed incoming data is processed by the U-Net-based autoencoder to produce a feature image. Depending on the network configuration, the feature map’s dimensions might change. The U-Net based feature extractor’s (autoencoder’s) general training configuration included the following parameters: a batch size of 64, 100 epochs, a patience value of 15, mean squared error (MSE) as the loss function, Adam as the optimizer, and MAE as the metric being tracked. The sample quantity and training period were also recorded. On the most recent feature map, the pixel-wise soft-max algorithm was used to calculate the loss function as well as the U-energy Net’s function. The soft-max function is defined as depicted in Equation (4):

(4) $$p_{n}\left(x\right)=exp(a_{n}\left(x\right))/(\sum_{J=1}^{N}exp(a_{j}\left(x\right)))$$

where $a_{n}\left(x\right)$ denotes the feature channel n activation at the pixel location xϵc, where $c\epsilon R^{2}$. In this instance, *N* stands for the overall number of courses, which will be 2 for each Unet. The total penalty is computed using $p_{l\left(x\right)}\left(x\right)$, where $l\left(x\right)$ is the point x ground truth.

Occasionally, this cost is calculated utilizing the weighted cross-entropy provided by Ronneberger et al. [23] as depicted in Equation (5):

(5) $$E=\sum_{x\in \varphi }w\left(x\right)\log_{p_{l(x)}}\left(x\right)$$

where w:φ→R refers to the weight map. The weight map, which is created to provide selected cells with more weight, is calculated using the following Equation (6):

(6) $$w\left(x\right)=w_{c}\left(x\right)+w_{0}.exp(-\frac{{\left(d_{1}\left(x\right)+d_{2}\left(x\right)\right)}^{2}}{{2\sigma }^{2}})$$

where *wc:* φ→*R* s is the weighting scheme for balancing class numbers, and d_1_: φ→ R and d_2_: φ→ R are the distances to the borders of the closest and second-closest cells, respectively.

The utilization of data supplementation, which enables a representationally larger collection of the training data, is another essential component of U-Net training. This study must create additional training examples by warping the present set of images when the training set is small (as it is with SpaceNet), as this will increase the model’s resilience. These anomalies are produced by a number of elastic deformations, such as horizontal flipping, breadth and height shift, shear, and zoom.

## 7. Feature Selection Using Hybrid Optimization Algorithm

Feature minimization is the process of reducing the number of input characteristics for a forecast model. This improves efficiency and lowers the processing expense of simulation. The feature optimization used in the hybridization architecture is covered in this section.

Propagation state: Every water wave will precisely spread during each cycle. Given the initial water wave x, the water wave that is spread through the propagation procedure will be D-sized, which is the measure of the maximal function F. The procedure shown in the following Equation (7) is used to carry out the propagation operation x:

(7) $$x^{\prime }\left(d\right)=x\left(d\right)+rand\left(-1,1\right).?L(d)$$

where $d\in D_{,}x\left(d\right)$ indicates the d−th dimension of the original water wave, x,x(d) is the d−th dimension of the propagated water wave, rand(−1,1) represents a uniformly distributed random number among [−1,1], δ shows the wavelength of the water wave, and L(d) refers the length of the d−th dimension of the search space. If the displacement surpasses *L(d)*, it will be arbitrarily assigned to a new location as shown in Equation (8):

(8) $$L\left(d\right)=lb\left(d\right)+rand.(ub\left(d\right)-lb\left(d\right))$$

where lb(d) and ub(d) are the lower and upper bounds of L(d), respectively, and rand is a random number generated between [0,1].

Following the dissemination process, the fitness value $f\left(x^{\prime }\right)$ is determined using the fitness function f.

Each water wave’s frequency will be adjusted after each repetition, in accordance with the Equation (9):

(9) $$\delta =(\delta .\alpha^{-f(x)}-f_{min}+\varepsilon )(f_{max}=f_{min}+\varepsilon )$$

where α represents the wavelength, and $f_{max}$ and $f_{min}$ refer to the maximum and minimum fitness values.

Refraction state: When water flows through precipitous mountain slopes with deep marine features, the waves are refracted. The wave height declines sequentially during transmission to mimic the energy loss of ocean waves and prevent search stagnation. Following the reflection of ocean waves, each dimension’s location is updated using Equation (10):

(10) $$x^{\prime }\left(d\right)=N(\frac{x^{*}\left(d\right)+x\left(d\right)}{2},\frac{\left|x^{*}\left(d\right)+x\left(d\right)\right|}{2})$$

where x* represents the ideal population size at the moment. The Gaussian distribution is denoted by N(μ,σ). Following the aforementioned action, hmax receives wave height x back, and the water wave wavelength is revised in accordance with Equation (11):

(11) $$\tau =\tau \frac{f(x)}{f(x)}$$

Prey encircling: The whale algorithm begins this step with the original top search agent. It is assumed that it has the best solution and is currently at/near the position of the target. As a result, the remaining agents update the position of the top search agent with their own locations. Equations (12) and (13) serve to express this process:

(12) $$D^{\prime }=\left|C^{\prime }.X^{\prime }\left(t\right)-X^{\prime }(t)\right|$$

(13) $${X*}^{\prime }\left(t+1\right)={X*}^{\prime }\left(t\right)-A^{\prime }.D^{\prime }$$

where $A^{\prime }$ and $C^{\prime }$ are coefficient vectors, and t denotes the current cycle. The location vector of the greatest solution thus far is ${X*}^{\prime }$ and its location vector. If a superior solution emerges, then ${X*}^{\prime }$ should be iteratively revised. These are the calculations for vectors *A′* in Equation (14) and *C*′ Equation (15):

(14) $$A^{\prime }=2.a^{\prime }.r^{\prime }-a^{\prime }$$

(15) $$C^{\prime }=2.r^{\prime }$$

where a′ falls from 2 to 0 over the number of repeats, and r is a random vector with a range of [0,1].

By simulating the target’s surrounding, this modeling enables any agent to update its position in the vicinity of the actual best solution. The movement through hypercubes will be assisted by the agents surrounding the most successful answer, and it is possible to further explore the search space in n dimensions.

Exploitation phase: This stage is also known as ‘bubblenet assaulting’, and it employs two strategies. The first strategy is the shrinking encircling mechanism, in which the value of $a^{\prime }$ in equation (3) is reduced, which also reduces the variation range of $A^{\prime }$ by $a^{\prime }$. This suggests that $a^{\prime }$ is arbitrarily inserted into ${[-a}^{\prime },a^{\prime }$], where over the course of refining, a falls from 2 to 0. The search agent’s new address can be located anywhere between the agent’s prior location and the optimal location at the moment due to the unpredictability of $A^{\prime }$ in the range of $\left[-1,1\right].$ The second strategy is the spiral updating position, which mimics humpback whales’ helix-shaped movement. A circle is formed between the positions of the whale and its target. This can be stated as following in Equation (16):

(16) $$X^{\prime }\left(t+1\right)=\left\{\begin{array}{cc} X{*}^{\prime }(t)=A^{\prime }.D^{\prime } & if\;p<0.6\\ D^{\prime }.e^{bl}.\cos\left(2\pi l\right)+X{*}^{\prime \left(t\right)} & if\;p\geq 0.5 \end{array}\right.$$

where p represents a random number in the range of [0,1].

Exploration phase: In order to persuade the search agent to depart from the reference cetacean, the *A*′ is assigned an arbitrary value from [−1,1]. As a result, ${-A}^{\prime }$ must either be higher than 1 or lower than −1. Additionally, a search agent that permits the WOA to conduct a worldwide search is chosen at random to refresh this position’s search agent. The algebraic expression for this research method is as showing in Equations (17) and (18):

(17) $$D^{\prime }=\left|C^{\prime }.X^{\prime }rand-X^{\prime }\right|$$

(18) $$X^{\prime }\left(t+1\right)=X^{\prime }rand-A^{\prime }.D^{\prime }$$

where $X^{\prime }rand$ is a randomly chosen whale from the current population, and the position is random.

Therefore, the text file containing these characteristics was used as the input file for the categorization algorithm. These steps were completed for a feature vectors file that was split. This study had 31 distinct profiles for each sample since each optimization technique had its own profile.

## 8. Classification Using DenseNet

**Figure 3:**
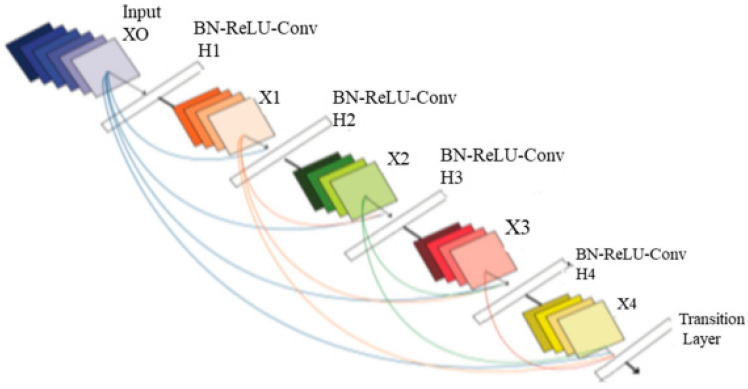
Architecture of DenseNet.

The method of classification involves grouping similar data types into groups. Numerous variables must be considered when choosing the best classifications. These elements include processing power, speed, and precision. The next stage is to forecast and group the information into the four tumor groups after choosing the best collection of characteristics. DenseNet can improve feature map transmission, solve the disappearing gradient issue, and minimize the number of parameters required. Figure 3 provides a general depiction of the suggested model’s architecture.

The four input channels of the suggested architecture’s input convolution layer are labeled with the following terms: dural metastasis (DM), leptomeningeal carcinomatosis (LC), miliary metastasis (MM), and intravascular carcinomatosis (IC). The primary benefit of this combination is the simultaneous processing of all four perspectives of the brain images. A convolution layer with a kernel dimension of 7 × 7 and a span of 2 constitutes each input layer in the DenseNet design. The initial dimension of the images is reduced to 112 × 112 × 3 by this convolution process. The input image consequently goes through a pooling layer with a limit of 3 × 3 and a cadence of 2 × 2. Thus, the convolution and pooling operations in the input layer lower the input image size to 56 × 56 × 3 prior to moving on to the packed block.

Transition layer: In the DenseNet model, straight links from any layer to all following layers form a distinct connectedness structure with other CNNs, which may further improve the flow of information between levels. The feature maps of all layers before it are given to the *l*-th layer as a consequence, and the following calculation is performed by Equation (19):

(19) $$x^{l}=H^{l}(\left[x^{0},x^{1},\ldots \ldots ,x^{l-1}\right])$$

in which l refers to the layer, $x^{1}$ represents the l-th layer’s outcome, and $\left[x^{0},x^{1},\ldots \ldots ,x^{l-1}\right]$ is the concatenation of the feature maps. 

The following highlights the primary distinction between the DenseNet and ResNet models. ResNet incorporates a skip-connection that uses an identity function to omit the non-linear transformations that calculated by Equation (20):

(20) $$x^{l}=H^{1}\left(x^{l}\right)+x^{l-1}$$

Dense Block Layer: In a system of four dense blocks, each layer responsible for creating a k-characteristic map following convolution while ensuring that the feature maps of each layer are of equal size. All the features from the levels are extracted using K convolution kernels. The network development rate, or number k, is referred to as a hyperparameter in DenseNet. To minimize processing and improve the effectiveness of the dense block, each dense layer obtains various data from earlier levels. The bottleneck layer (1 × 1 convolution layer between batch normalization, ReLU, and a 3 × 3 convolution layer) is utilized internally by the dense block.

Output Classification Layer: The suggested architecture’s output layer includes a unique average pooling layer for each channel to derive useful characteristics. The extracted characteristics are provided to the particular dense layer after being smoothed by the flatten layer. Two combination blocks are used to combine the MBD characteristics obtained from the four channels. The final combination block connects all of the characteristics of the suggested approach. All of the characteristics are accepted by the three dense layers before the categorization layer is given the output of the three dense layers. Table 1 displays the technological specifications of the suggested design.

## 9. Performance Analysis

For the purpose of analysis, accuracy, precision, recall and F1-score were selected as the parameters. The suggested DenseNet_ HybWWoA is compared with three standard methods, namely the Harris Hawks optimized convolution network (HHOCNN) [12] and ResNet-152 [13], based on these parameters.

The model’s capacity to render a general forecast is shown by its accuracy. Metastases can be predicted to be present or absent using true positive (TP) and true negative (TN) results. False positive (FP) and false negative (FN) results represent the model’s inaccurate forecasts. Accuracy is calculated using the following Equation (21).

(21) $$Accuracy=\frac{TP+TN}{TP+FP+FN+TN}$$

Precision: The rate of precision is the ratio of the number of true positive samples to the total number of positive samples. In other words, precision represents the proportion of the predicted positive samples that are actually positive when metastases are present. Precision is defined in (22):

(22) $$Precision=\frac{TP}{TP+FP}$$

Recall: The sensitivity computation does not take ambiguous test results into consideration, as a test cannot be replicated, and all uncertain samples should be omitted from the analysis. This affects the capacity to correctly identify metastases in a dataset. t is calculated using the following Equation (23):

(23) $$Recall=\frac{TP}{TP+FN}$$

F1-score: The F1-score is used to assess the accuracy of predictions. It represents the weighted sum of memory and accuracy. The highest possible value is 1, and the worst value is 0. The F1-score is computed without taking TNs into account. Table 2 shows the performance analysis of accuracy for different methods.

Figure 3 depicts the accuracy evaluation. The suggested DenseNet_ HybWWoA is evaluated against the conventional HHOCNN and ResNet-152. The x-axis and y-axis show the number of epochs and the values obtained as a percentage, respectively. These results indicate that while HHOCNN and ResNet-152 achieved 85.2% and 86.4% accuracy, respectively, the proposed DenseNet_ HybWWoA obtained 98.5% accuracy, which was 13.07% and 12.27% higher than HHOCNN and ResNet-152, respectively. Precision performance analysis for several approaches is shown in Table 3.

**Figure 4:**
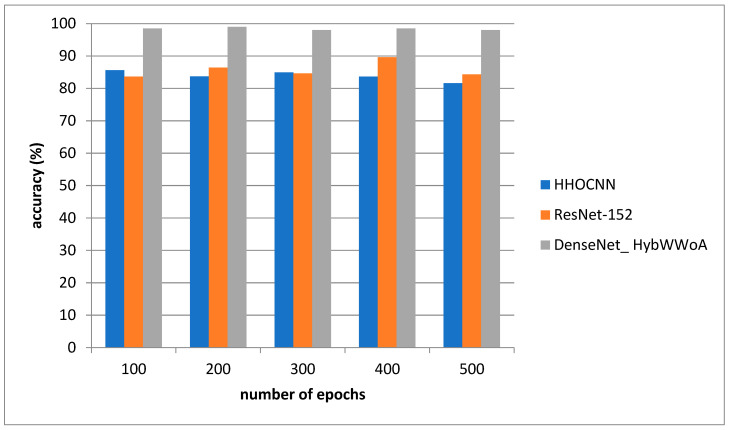
Analysis of accuracy.

Figure 4 depicts the precision evaluation. The comparison is performed between the existing HHOCNN and ResNet-152 with the proposed DenseNet_ HybWWoA. The x-axis and y-axis show number of epochs and the values obtained as a percentage, respectively. While HHOCNN and ResNet-152 achieved 87.5% and 86% precision, respectively, the proposed DenseNet_ HybWWoA obtained 92.1% precision, which was 7.2% and 6.3% superior to HHOCNN and ResNet-152, respectively. Table 4 shows the performance analysis of recall for severl approaches.

**Figure 5:**
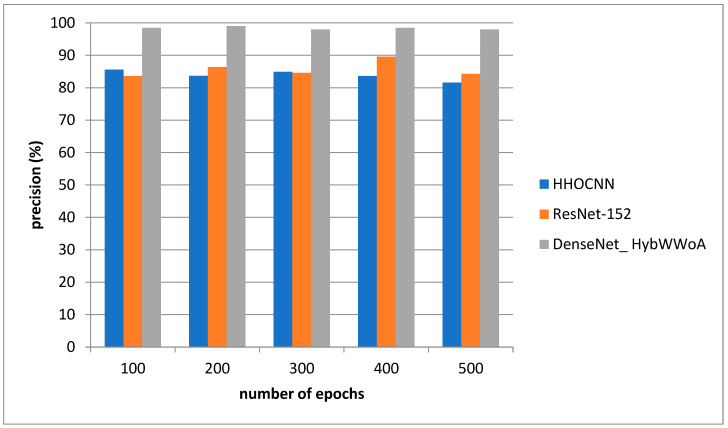
Analysis of precision.

Figure 5 depicts the recall evaluation. The comparison is performed between the existing HHOCNN and ResNet-152 and the proposed DenseNet_ HybWWoA. The x-axis and y-axis show number of epochs and the values obtained as a percentage, respectively. The existing HHOCNN and ResNet-152 achieved 78.3% and 86.1% recall, respectively, while the suggested DenseNet_ HybWWoA obtained 96.3% recall, which was 18% and 10.2% superior to HHOCNN and ResNet-152, respectively. Comparative study of the effects of different approaches on F1-scores is demonstrated in Table 5.

**Figure 6:**
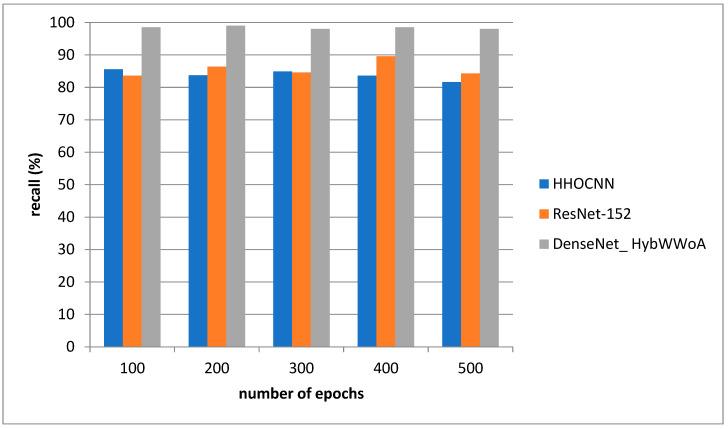
Analysis of recall.

Figure 6 depicts the F1-score evaluation. The comparison is performed between the existing HHOCNN and ResNet-152 with the proposed DenseNet_ HybWWoA. The x-axis and y-axis show the number of epochs and the values obtained as a percentage, respectively. HHOCNN and ResNet-152 achieved 85% and 87% F1-score, respectively, while the proposed DenseNet_ HybWWoA obtained 97% F1-score, which was 12% and 10% superior to HHOCNN and ResNet-152, respectively.

## 10. Discussion and Conclusions

Overall, the novel DenseNet_ HybWWoA algorithm successfully proved to be superior to other contemporary imaging methodologies for the radiological evaluation of the T1-weighted contrast-enhanced MRI images investigated in this study. Table 6 clearly outlines the positive influence of adopting such a combinatory algorithm approach across all major MRI performance parameters in comparison to the HHOCNN and ResNet-152 methodologies. Concerning the accuracy of MRI analytical methodologies, DenseNet_ HybWWoA was found to be 13.3% superior to HHOCN and ResNet-152. Other striking results included the superiority of this novel hybrid algorithm by 18% in recall performance when compared to the HHOCN methodology alone.

The clinical advantages of the DenseNet_ HybWWoA algorithm include a reduction in the incidence rate of false positive and false negative results in radiologist assessments for cerebral MRI images. This increased accuracy and precision in MRI image analysis will certainly contribute to a reduction of differential diagnoses—particularly concerning brain metastatic lesions—and also provide a more accurate clinical status for the clinical oncologist, allowing them to perform more bespoke therapeutic strategies that are based on crucial imaging data. The overall comparative analysis is presented in Table 6. 

Furthermore, the DenseNet_ HybWWoA can eventually lead to the adoption of a more cost-effective MRI analytical protocol, consequently relieving a degree of financial burden upon both medical institutions/clinics and patients.

This study’s findings suggest that the DenseNet_ HybWWoA design categorizes provided brain images as normal or malicious. The developed network performed better than pre-existing, pre-trained networks when assessed on T1-weighted contrast-enhanced magnetic resonance images. The 10-fold cross-validation method yielded optimal findings for the record-wise cross-validation, with an accuracy of 98.5%. Future work will focus on including ensemble-based classification methods to improve overall accuracy and speed.

## Figures and Tables

**Figure 7:**
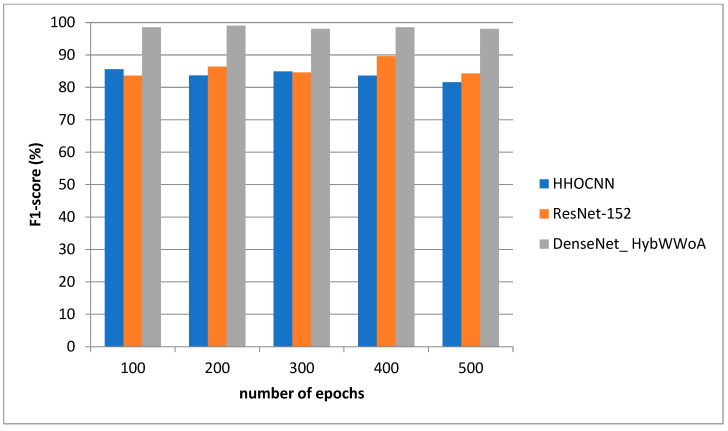
Analysis of F1-scores.

**Table 1:** Technical specifications of DenseNet.

Layer/Channel	Output Size/Channel
Convolution	Kernel 8 × 8 s
pooling	4 × 4 max pooling
Dense block-1	5 × 5 conv × 4
Transition 1	Batch normalization 2 × 2 convolution layer
Dense block-2	1 × 1 × conv
Transition 2	Batch normalization 4 × 4 convolution layer
Dense block-3	5 × 5 × conv
Transition 3	Batch normalization 6 × 6 convolution layer
Dense block-4	7 × 7 × conv
Transition 4	Batch normalization 8 × 8 convolution layer
Classification layer	7 × 7 global averages

**Table 2:** Performance analysis of accuracy for differing methods (see Figure 4).

Number of Epochs	HHOCNN	ResNet-152	DenseNet_ HybWWoA
100	85.6	83.6	98.5
200	83.7	86.4	99
300	84.9	84.6	98
400	83.6	89.6	98.5
500	81.6	84.3	98

**Table 3:** Performance analysis of precision for differing methods (see Figure 5).

Number of Epochs	HHOCNN	ResNet-152	DenseNet_ HybWWoA
100	82.6	87.4	91.3
200	84.9	86.5	92.4
300	87.3	87.2	92.5
400	82.5	86.3	92.8
500	84.6	87.9	93.7

**Table 4:** Performance analysis of recall for differing methods (see Figure 6).

Number of Epochs	HHOCNN	ResNet-152	DenseNet_ HybWWoA
100	78	86.4	97.45
200	77	87.4	96.2
300	77.9	86.5	98.4
400	78.4	85.3	96.9
500	78.4	82.6	94.8

**Table 5:** Performance analysis of F1-scores for differing methods (see Figure 7).

Number of Epochs	HHOCNN	ResNet-152	DenseNet_ HybWWoA
100	81.8	84.6	97.65
200	86.4	87.4	96.58
300	87.4	84.5	98.32
400	84.6	87.5	97.48
500	82.6	86.4	98.25

**Table 6:** Overall comparative analysis.

Parameters	HHOCNN	ResNet-152	DenseNet_ HybWWoA
Accuracy (%)	85.2	85.2	98.5
Precision (%)	87.5	86	92.1
Recall (%)	78.3	86.1	96.3
F1-score (%)	85	87	97

## Data Availability

The dataset used in the experiments is publicly available from Figshare (https://figshare.com/articles/dataset/brain_tumor_dataset/1512427).
